# ASSOCIATION BETWEEN WAIST-BASED ANTHROPOMETRIC INDICES AND MILD COGNITIVE IMPAIRMENT FROM THE KFACS

**DOI:** 10.1093/geroni/igae098.3798

**Published:** 2024-12-31

**Authors:** Daehyun Lee, Hyung Eun Shin, Heeeun Jung, Jae Young Jang, Hyunjin Cho, Nahyun Lim, Chang Won Won, Miji Kim

**Affiliations:** Kyung Hee University, Seoul, Republic of Korea; Kyung Hee University, Seoul, Republic of Korea; Kyung Hee University, Seoul, Republic of Korea; Kyung Hee University, Seoul, Republic of Korea; Kyung Hee University, Seoul, Republic of Korea; Kyung Hee University, Seoul, Republic of Korea; Kyung Hee University, Seoul, Republic of Korea; Kyung Hee University, Seoul, Republic of Korea

## Abstract

While several studies have explored the association between obesity and cognitive function in older adults, definitive evidence linking waist-based anthropometric indices to impaired cognitive function remains lacking. Our study aimed to investigate the association between the waist-based anthropometric indices and mild cognitive impairment (MCI) in older adults according to sex. We conducted a retrospective study on 1,192 community-dwelling older adults (51.8% women; mean age 75.1±3.6 years) from the Korean Frailty and Aging Cohort Study during a 6-year follow-up. The waist-based anthropometric indices at baseline include waist circumference (WC), waist-to-height ratio (WHtR), waist-to-weight ratio (WWR), and weight-adjusted-waist index (WWI). These indices were calculated as follows: WHtR= WC (cm) / height (cm), WWR= WC (cm) / body weight (kg), and WWI= WC (cm) /√(body weight (kg)). MCI incidence was measured from Wave 2 to 4, with MCI defined as Mini-Mental State Examination< 24. Multivariate logistic regression analysis was utilized to examine the association between these waist-based anthropometric indices and MCI, and restricted cubic spline curve was used to assess the potential nonlinear relationship. The incidence of MCI was 21.1% in men and 34.1% in women during 6-year follow-up (p< 0.01). The highest WWI quintile was significantly associated with MCI only in men (odds ratio: 4.49, 95% confidence interval: 1.39–14.47) in the fully adjusted models. However, no significant associations were found in WC, WHtR, and WWR. Our findings suggest that WWI is more strongly associated with MCI compared to other waist-based anthropometric indices in men, proposing its potential as a clinical indicator for MCI.

